# Introducing Sedatic Hunger: Eating To Survive, Not to Savor

**DOI:** 10.1007/s13668-025-00677-6

**Published:** 2025-06-20

**Authors:** Sedat Arslan, Hande Öngün Yilmaz, Emre Ciydem

**Affiliations:** 1 Department of Nutrition and Dietetics, Bandirma Onyedi Eylul University, Balikesir, Turkey; 2 Nursing Department, Faculty of Health Science, Bandirma Onyedi Eylul University, Balıkesir, Turkey

**Keywords:** Eating Behavior, Necessity Eating, Survival Eating, Sedatic Hunger

## Abstract

**Purpose of Review:**

This review introduces *Sedatic Hunger* as a distinct and underexplored form of hunger. Unlike physiological or hedonic hunger, Sedatic Hunger refers to a neutral, function-oriented motivation to eat—driven by biological need without the pursuit of taste, pleasure, or emotional satisfaction. The purpose of this review is to conceptualize Sedatic Hunger, differentiate it from other hunger types, and highlight its clinical and societal relevance.

**Recent Findings:**

Emerging clinical observations suggest that individuals with depression, restrictive dietary habits, or those under chronic stress may experience Sedatic Hunger. These individuals consume food purely for energy, with limited sensory or emotional engagement. The article outlines preliminary efforts to develop the *Sedatic Hunger Scale (SHS)* to assess this construct, aiming to quantify how biological, psychological, and cultural factors intersect in this form of eating behavior.

**Summary:**

Sedatic Hunger expands our understanding of human motivations for eating by focusing on biologically driven but emotionally neutral food intake. Its recognition may improve approaches in mental health, dietary counseling, and nutrition science. Future research directions include identifying biological markers, evaluating the effects of sensory deprivation, and exploring cross-cultural differences in the manifestation of Sedatic Hunger.

## Introduction: The Emergence and Need for the Concept of Sedatic Hunger

In recent decades, the study of eating behaviors has increasingly illuminated the intricate relationship between physiological needs and psychological motivations [1, 2]. The field has made considerable strides in dissecting hunger into types, with *physiological hunger*—driven by the body’s energy requirements—and *hedonic hunger*—motivated by sensory pleasure and emotional satisfaction—receiving the bulk of attention [3, 4]. These established frameworks offer vital insights into why we eat, underscoring the dynamic role that both body and mind play in feeding behavior [5]. However, as our understanding has deepened, it has become clear that not all eating behavior neatly aligns with pleasure or energy-based motivation.

In particular, decades of observational research and clinical practice have frequently overlooked a phenomenon we have identified: individuals who engage in eating purely as a functional act, devoid of sensory engagement or pleasure-seeking. This form of eating challenges conventional categorizations, suggesting a hunger driven solely by biological necessity without the pursuit of taste or satisfaction. We term this unique phenomenon Sedatic Hunger. Further details on its definition and core characteristics are discussed in the following section.

### Definition of Sedatic Hunger

The term “Sedatic Hunger” is inspired by the concept of sedation, embodying a neutral, subdued approach to eating, in which sensory enjoyment plays no role. Interestingly, the term also humorously nods to our first author’s name, Sedat, creating both a personal and academic alignment with this concept. Sedatic Hunger thus represents a practical, duty-focused view of eating, driven solely by the need to fulfill physiological requirements. Unlike hedonic hunger, which actively seeks sensory pleasure, Sedatic Hunger underscores the purely functional aspect of hunger, where food is regarded as mere fuel.

This article introduces Sedatic Hunger as a distinct type of hunger characterized by eating without the pursuit of pleasure or satisfaction. Unlike hedonic hunger, which actively seeks enjoyment and sensory stimulation, Sedatic Hunger is proposed here as a hunger type that prioritizes only the body’s biological necessity. In this form of hunger, the taste, texture, or any potential pleasure of food is irrelevant; the primary goal is simply to meet the body’s energy and nutrient needs. Unlike physiological and hedonic hunger, which include elements of satisfaction and sensory enjoyment, Sedatic Hunger embodies a neutral approach to eating, where food’s sensory qualities are irrelevant, and eating is treated solely as a functional duty.

To further clarify the distinctive characteristics of Sedatic Hunger, Table 1 presents specific criteria that differentiate it from other types of hunger. Each criterion highlights the unique lack of sensory or psychological engagement associated with Sedatic Hunger, focusing on the purely functional nature of eating as seen in individuals who experience this form of hunger. These criteria provide a structured framework for identifying Sedatic Hunger in research and clinical contexts.

**Table 1:** Criteria defining sedatic hunger

Criterion	Explanation
**Absence of Taste and Sensory Pleasure Seeking**	In Sedatic Hunger, the taste, texture, or sensory enjoyment of food holds no significance. Eating is seen purely as a necessary act, rather than a source of pleasure or interest.
**Indifference to Sensory Stimuli**	Visual appeal, aroma, or presentation of food does not trigger Sedatic Hunger. Individuals remain indifferent to these sensory cues, focusing solely on physiological needs.
**Lack of Satisfaction or Enjoyment Post-Meal**	After eating, there is no sense of pleasure or satisfaction. Food is not perceived as a gratifying experience, highlighting a sense of duty rather than enjoyment in eating.
**Independence from Psychological or Emotional Needs**	Eating is not motivated by psychological comfort or emotional satisfaction but purely by the body’s energy requirements. Emotional states do not influence Sedatic Hunger.
**Preference for Accessibility**,** Caloric Sufficiency**,** and Practicality Over Sensory Engagement**	Those experiencing Sedatic Hunger often select foods based on accessibility, practicality, or caloric sufficiency, regardless of sensory or nutritional value.
**Disregard for the Social or Cultural Value of Food**	The act of eating is independent of social or cultural contexts. Food is seen as merely a physiological requirement, without the importance of eating in a social setting.
**Neutral or Detached Approach to Eating**	Individuals exhibit a neutral or detached attitude toward food. Eating is viewed as a “task” or “obligation,” with minimal emotional engagement.

## Comparison with Existing Types of Hunger: Distinguishing Sedatic Hunger

Over the past few decades, nutritional science has methodically classified hunger types to better understand the myriad motivations behind eating behavior. Physiological hunger and hedonic hunger remain the two principal types in contemporary literature, each shedding light on distinct drives. However, emerging observational data and clinical insights suggest that these classifications may overlook a nuanced yet significant form of hunger: Sedatic Hunger. In this section, we will draw comparisons to delineate Sedatic Hunger and highlight its unique characteristics within the spectrum of hunger types.

### Physiological Hunger

Physiological hunger, as documented extensively in the literature, is primarily a biological response to the body’s energy needs [3, 6, 7]. This hunger type is often accompanied by an intrinsic satisfaction once satiation is achieved, frequently involving a preference for certain flavors or textures that enhance the eating experience. When physiological hunger is addressed, a person may experience not only physical relief but also a subtle sense of pleasure, aligning the body’s caloric requirements with a degree of sensory enjoyment [8].

### Hedonic Hunger

In contrast, hedonic hunger represents a form of eating that is largely detached from the body’s immediate energy needs. Driven by sensory appeal, comfort, or psychological satisfaction, hedonic hunger is fueled by the pursuit of pleasure rather than necessity [4, 9]. The food choices made under hedonic hunger are highly influenced by flavor, aroma, texture, and appearance. This type of hunger may arise even in a state of physiological satiety, where the individual consumes food solely for the rewarding sensory experience it provides.

### How Sedatic Hunger Differs

While physiological hunger involves satisfaction of hunger and energy needs, and hedonic hunger seeks sensory pleasure; Sedatic Hunger exists as a duty-oriented, neutral approach to eating. Individuals experiencing Sedatic Hunger eat strictly to meet bodily needs, indifferent to sensory qualities or satisfaction. In this context, food is merely fuel, and the act of eating lacks the engagement seen in other hunger types, underscoring Sedatic Hunger’s unique position within the spectrum of eating behaviors.

### Comparison of Sedatic Hunger with Other Eating Approaches

The concept of Sedatic Hunger offers a novel perspective in the field of eating behaviors by addressing dimensions that are often overlooked in existing frameworks. To enhance the scholarly value of this concept, it is essential to delineate its relationship with and distinctions from existing approaches, namely Internally Regulated Eating, Intuitive Eating, and Epicurean Eating. This section provides a structured comparison to highlight the unique contributions of Sedatic Hunger to the literature.

### Sedatic Hunger and Internally Regulated Eating

Internally Regulated Eating refers to eating in response to internal physiological signals, such as hunger and satiety, rather than external cues [10]. A detailed comparison between Sedatic Hunger and Internally Regulated Eating is provided in Table 2.

**Table 2:** Comparison of sedatic hunger and internally regulated eating

Aspect	Internally Regulated Eating	Sedatic Hunger
**Focus**	Responds to physiological signals	Responds solely to physiological needs
**Psychological Satisfaction**	May include satisfaction or relief	Excludes any psychological satisfaction
**Food Selection**	Allows selection of enjoyable foods	Focuses entirely on functionality

### Sedatic Hunger and Intuitive Eating

Intuitive Eating rejects diet culture, encourages individuals to honor hunger signals, build a positive relationship with food, and view eating as a pleasurable experience [11]. Table 3 compares the core characteristics of Intuitive Eating and Sedatic Hunger, highlighting their conceptual differences.

**Table 3:** Comparison of sedatic hunger and intuitive eating

Aspect	Intuitive Eating	Sedatic Hunger
**Focus**	Bodily signals and sensory experiences	Solely physiological needs
**Pleasure in Eating**	Central to the concept	Completely absent
**Approach**	Holistic and enjoyment-driven	Functional and obligatory

### Sedatic Hunger and Epicurean Eating

Epicurean Eating emphasizes deriving pleasure from the aesthetic and sensory experience of eating, often prioritizing quality over quantity [12]. Table 4 provides a comparative overview of Epicurean Eating and Sedatic Hunger, illustrating the contrast between sensory-focused and function-focused approaches to food.

**Table 4:** Comparison of sedatic hunger and epicurean eating

Aspect	Epicurean Eating	Sedatic Hunger
**Focus**	Sensory delight and pleasure	Excludes sensory engagement
**Nature of Eating**	Aesthetic and pleasure-driven	Detached and functional
**Cultural Context**	Strongly tied to cultural and sensory values	Detached from cultural or sensory values

### The Unique Contribution of Sedatic Hunger

Sedatic Hunger offers a unique lens to understand eating behaviors that are disconnected from sensory and psychological drivers. This concept fills a critical gap in the literature by defining eating behavior solely as a physiological necessity. Unlike existing approaches, Sedatic Hunger emphasizes the neutral and obligatory nature of food consumption.

The structured comparison provided herein underscores the distinctive features of Sedatic Hunger relative to other well-established eating frameworks. By conceptualizing eating as a strictly functional act, Sedatic Hunger enriches the discourse on eating behaviors, paving the way for future research and clinical innovations.

### Economic Dimensions of Sedatic Hunger

Sedatic Hunger may often emerge in contexts where economic hardship limits access to a variety of enjoyable foods, as seen in cases of food insecurity [13, 14]. This reality highlights how socioeconomic factors intersect with eating behaviors, transforming food into a necessary but unenjoyable routine. In many societies, economic factors play a significant role in shaping individuals’ eating behaviors and perceptions of food [15, 16]. Food insecurity, defined as limited or uncertain access to adequate food due to financial constraints, forces individuals to prioritize basic sustenance over sensory enjoyment [17]. In the context of food insecurity, individuals often prioritize functional, accessible, and energy-dense foods to meet basic caloric needs. This necessity-driven approach to eating is shaped by external constraints such as financial limitations and limited food availability, rather than a deliberate focus on nutritional quality [17–20]. This economic context offers a unique lens for understanding Sedatic Hunger, where food is reduced to a means of survival, often leading to the consumption of inexpensive, energy-dense options, with little regard for sensory satisfaction or nutritional quality.

While Sedatic Hunger often emerges in economically disadvantaged settings due to limited food access and financial constraints, the food choices in this context are typically dictated by affordability and practicality. Importantly, Sedatic Hunger is not exclusive to food-insecure populations. Individuals with high socioeconomic status may also exhibit Sedatic Hunger, albeit driven by factors such as fast-paced lifestyles, performance-oriented eating, or cultural pressures around diet and body image. This demonstrates that Sedatic Hunger is a multidimensional phenomenon influenced by diverse socioeconomic and psychological dynamics, extending beyond food insecurity to encompass broader behavioral and cultural patterns.

The study of eating behaviors has long been anchored in examining pleasure and satisfaction as primary motivators. Most research in this domain has focused on the sensory and psychological aspects of eating, emphasizing how pleasure-driven motivations shape food choices and consumption patterns [21–25]. While hedonic hunger represents the apex of this sensory-seeking motivation, even physiological hunger is often associated with a degree of satisfaction or pleasure upon fulfillment. However, these studies tend to overlook a less conspicuous form of hunger that arises solely from a biological requirement, one that is void of sensory engagement or psychological fulfillment—a form we have termed Sedatic Hunger.

### Literature Gaps

While extensive work has explored how sensory pleasure and emotional satisfaction influence eating behaviors, there remains a significant gap concerning individuals who approach food as merely a functional necessity [25–27]. This oversight implies a limited understanding of the full spectrum of eating motivations, neglecting those who do not seek or consider sensory enjoyment in their relationship with food. Sedatic Hunger thus fills this gap by acknowledging that eating is a purely obligatory act entirely detached from psychological or sensory gratification for some. This perspective opens up new avenues for research, encouraging a broader understanding of eating behaviors that move beyond the pleasure-seeking paradigm [28]– [29].

### Comparison with Existing Literature

The dichotomy between hedonic and physiological hunger has largely shaped our understanding of why we eat. Studies on hedonic hunger underscore how sensory pleasure drives food consumption, even in the absence of physical hunger, highlighting the role of taste, aroma, and other sensory attributes in creating a desire to eat [26, 30–32]. On the other hand, research on physiological hunger addresses hunger as a biological necessity, often with a sense of satisfaction upon satiation. Sedatic Hunger extends beyond these established categories, embodying a form of hunger where eating is strictly a biological function, lacking any accompanying sense of pleasure or enjoyment. Here, food is reduced to its most fundamental role—as fuel for the body—suggesting a unique interpretation of hunger that challenges traditional motivations behind eating behaviors [33, 34].

Sedatic Hunger provides a novel framework through which we can study eating behaviors that fall outside the pleasure-seeking or satisfaction-driven models. By addressing this gap, Sedatic Hunger contributes to a more comprehensive understanding of the motivations behind food consumption and offers potential insights into the nutritional needs and dietary habits of individuals who view eating as a non-sensory, purely functional act.

## Why Sedatic Hunger Matters: Theoretical and Practical Implications

Sedatic Hunger provides a novel framework in the study of eating behaviors, addressing a form of hunger that transcends traditional physiological and hedonic models. By recognizing this distinct phenomenon, we open pathways to explore non-pleasure-driven eating behaviors, which have been largely overlooked in the current literature. This concept is particularly valuable in understanding how individuals approach eating in contexts where sensory satisfaction or emotional fulfillment are absent, highlighting a unique dimension of human interaction with food.

Understanding Sedatic Hunger is particularly important in clinical contexts, where it can help explain behaviors seen in individuals experiencing depression, restrictive diets, or economic hardship. These individuals often approach eating as a purely functional act, detached from sensory or emotional engagement. Additionally, in societal contexts, Sedatic Hunger provides a lens to explore how food insecurity or fast-paced lifestyles shape indifferent eating patterns. By examining this phenomenon, we can better address nutritional challenges and support individuals whose food interactions are shaped by necessity rather than desire.

By recognizing Sedatic Hunger as a unique phenomenon, we can expand our understanding of human motivations for eating, bridging theoretical insights with practical applications. This concept not only enriches the field of nutrition science but also offers a foundation for developing personalized interventions in both clinical and societal contexts.

### Implications for Eating Behaviors

In societies where food is often culturally significant and celebrated as a source of pleasure and connection, understanding those who approach eating merely as a necessity is essential for fostering inclusivity in dietary studies [21, 24, 31, 33–37]. Sedatic Hunger sheds light on cases where individuals treat eating as a mandatory task, devoid of enjoyment or preference, framing eating behaviors as a responsibility rather than an experience. Such a framework allows us to better understand and support people for whom food lacks social or sensory appeal, and it raises important questions about how this outlook might shape their nutritional intake, food choices, and overall health [38–40].

### Factors Contributing To State-Driven Sedatic Hunger

From a clinical perspective, Sedatic Hunger offers a framework that could enhance our understanding of behaviors often associated with conditions like appetite loss, depression, or monotonous eating patterns. The relationships between depression, anhedonia, and appetite are complex and cyclical in nature. Depression often leads to significant changes in appetite [41]. While some individuals may experience appetite loss and weight loss, others may exhibit increased appetite and emotional eating behaviors, as seen in conditions like atypical depression [42]. Anhedonia, the inability to derive pleasure from previously enjoyable activities, including eating, can reduce a person’s motivation to eat or weaken the sense of reward derived from food consumption. These changes in appetite can affect the severity of depression, while poor eating habits may further exacerbate depressive symptoms [41, 43]. On a biological level, imbalances in neurotransmitters such as serotonin and dopamine can disrupt appetite regulation [41, 44]. Furthermore, dysfunctions in the hypothalamic-pituitary-adrenal (HPA) axis may alter the levels of appetite-regulating hormones such as ghrelin and leptin [45). The dopamine system, directly linked to anhedonia and reward sensation, may also contribute to a diminished sense of pleasure from food consumption when impaired [41]. Psychological and environmental factors play a crucial role in these dynamics as well. Social isolation caused by depression may disrupt regular eating patterns, while stress factors can exacerbate both depression and appetite-related issues [41, 45].

Sedatic Hunger can manifest in certain individuals and various contexts beyond medical conditions such as depression. For example, research indicates that prolonged adherence to monotonous or restrictive diets can lead individuals to perceive eating merely as a necessity [46–48]. A study by Sørensen et al. [49] found that monotonous diets reduce sensory satisfaction, transforming eating into an activity aimed solely at fulfilling physiological needs.

Moreover, a study involving workers in the technology sector revealed a relationship between work pressure, fatigue, and unhealthy eating behaviors [50]. Similarly, research on office workers identified unhealthy eating patterns, irregular meals, and skipping meals as contributing to imbalanced and insufficient nutrition [51]. These findings suggest that individuals with heavy workloads may prioritize quick, functional eating over sensory enjoyment due to time constraints and occupational demands [50, 51].

Additionally, a study investigating factors affecting workplace eating habits reported that food insecurity may compel individuals to focus solely on meeting physiological needs, leading to a disconnection from the sensory or emotional aspects of eating [52]. Research involving university students found that those experiencing high levels of perceived stress were more likely to engage in unhealthy dietary behaviors, such as consuming convenience foods [53]. These findings suggest that stressed individuals may adopt functional eating behaviors to maintain energy levels [53]. Similarly, a study on Belgian university students examining the cultural and social influences on eating behaviors revealed that factors such as food availability, affordability, and practicality outweighed sensory enjoyment in their food choices [54].

Another context where Sedatic Hunger may arise is among military personnel. A study on the eating behaviors of U.S. soldiers and their relationship with body composition, physical fitness standards, and physical performance reported that habitual dining, fast eating, and neglect of satiety signals led to prioritizing functional meal choices over sensory satisfaction [55]. These behaviors are shaped by environmental requirements such as time constraints, physical fitness standards, and nutritional education, transforming eating into a functional activity focused on energy intake rather than sensory reward [55].

Premenopausal women are another group where Sedatic Hunger can emerge. A study examining the relationship between perceived social isolation, brain reactivity to food, eating behaviors, obesity, and psychological symptoms found that women living alone exhibited lower diet quality and a greater tendency to consume processed or ready-to-eat foods [56]. These results indicate that eating behaviors in socially isolated women may become more functionally oriented toward energy intake.

Hospitalized patients represent another group prone to Sedatic Hunger. The consumption of hospital meals, often standardized to meet nutritional guidelines, is characterized by a lack of sensory satisfaction, leading to perceptions of blandness and lack of variety [57]. Supporting this, a study exploring the nutritional experiences of hospitalized patients found that factors such as the hospital environment, catheter bags, and intravenous catheters negatively impacted patients’ feeding processes and reduced their desire to eat [58].

These findings demonstrate that Sedatic Hunger can arise in various contexts, highlighting the need for a nuanced understanding of functional eating and its implications for both mental and physical health. In such cases, food may cease to offer comfort or enjoyment, being perceived instead as an obligation. This concept opens up new opportunities to explore how purely functional eating may impact mental and physical health. For example, individuals experiencing Sedatic Hunger may show distinct physiological responses or psychological tendencies that differ from those who eat for pleasure. Recognizing these patterns can aid clinicians in devising more targeted approaches, whether in addressing loss of appetite due to psychological conditions or developing dietary interventions for patients who perceive food as merely a means to an end [59–61].

Ultimately, Sedatic Hunger underscores the need for a more nuanced approach to nutritional counseling, therapy, and research, one that acknowledges eating as a multi-dimensional behavior influenced by a variety of personal, physiological, and psychological factors. By integrating Sedatic Hunger into our understanding of eating behaviors, we can provide more comprehensive care and tailored strategies that resonate with those who view food primarily as a biological necessity.

It is important to differentiate Sedatic Hunger from depression-related appetite loss and disordered eating behaviors such as binge eating or restrictive patterns. While depression and certain eating disorders involve alterations in appetite and reward perception, these changes are often pathological, involuntary, and symptomatic of emotional or neurobiological dysregulation. In contrast, Sedatic Hunger is not necessarily linked to dysfunction or distress. It represents a neutral, functional eating state that may arise in both healthy and clinical populations. Rather than being driven by emotional states or body image concerns, Sedatic Hunger is defined by an emotionally detached and task-oriented approach to eating, where food serves as fuel with minimal sensory or psychological engagement.

### Application Examples of Sedatic Hunger in Clinical and Societal Contexts

To fully appreciate the relevance of Sedatic Hunger in real-world settings, it is valuable to consider how this phenomenon manifests in various clinical and societal contexts. Sedatic Hunger, as a form of hunger detached from pleasure or sensory engagement, is particularly observable in situations where individuals experience a neutral or indifferent approach to food due to psychological or lifestyle factors. Incorporating practical examples or case illustrations can enhance our understanding of Sedatic Hunger’s impact, especially in fields such as mental health and nutritional counseling.

One notable example is found in individuals experiencing depression. Clinically, depression often leads to a reduced desire for previously enjoyed activities, including eating. For some, food becomes a mere necessity, stripped of its usual comfort or pleasure, embodying characteristics consistent with Sedatic Hunger. These individuals may eat solely to sustain themselves, displaying an indifference to taste, aroma, or meal variety. A similar pattern can be observed in patients undergoing monotonous or restrictive diets for health or weight management reasons, where food selection is limited, and the act of eating becomes routine, devoid of excitement or enjoyment. Over time, the lack of diversity and sensory appeal can lead to a functional, duty-based eating approach—essentially, Sedatic Hunger.

## Case Study Illustration

### Case 1

Consider a 45-year-old male patient diagnosed with chronic depression. In clinical interviews, he describes his relationship with food as “mechanical.” He eats the same basic meals each day, expressing no interest in taste or variety. When probed about his food choices, he responds that eating is “just something to get through the day.” Here, Sedatic Hunger provides a framework for understanding his experience, highlighting how psychological conditions can influence one’s approach to food, transforming it into a task devoid of sensory or emotional engagement.

These examples illustrate Sedatic Hunger’s potential presence in both clinical settings, such as mental health or dietetics, and broader societal behaviors. They underscore the need for a nuanced approach to nutritional counseling and mental health support, particularly for individuals who view eating as an obligation rather than an enjoyable experience. By considering Sedatic Hunger in these contexts, practitioners may better address the unique needs of patients and clients whose food interactions are shaped by function rather than fulfillment.

### Case 2

Consider K.Y. a university student living in a dormitory where he relies on the student cafeteria for his meals, which are provided free of charge. While this service helps him manage his limited budget, the food served is consistently unappealing; he describes the taste as bland and finds the variety lacking. Although he has no financial burden for these meals, K.Y. also has no feasible options to access food that might offer enjoyment or satisfy his preferences. As a result, he consumes the cafeteria meals out of necessity, viewing them simply as a way to “get by” rather than as an enjoyable activity.

In this scenario, Sedatic Hunger offers a useful framework for understanding K.Y.’s experience, emphasizing how external factors—such as financial constraints and limited access to appealing food—can transform eating into a purely functional task, devoid of sensory or emotional engagement. This example highlights the potential presence of Sedatic Hunger in societal settings and underscores the importance of recognizing such dynamics in nutritional counseling. By considering Sedatic Hunger in these contexts, practitioners may better support individuals whose food interactions are shaped by necessity rather than fulfillment, particularly in structured environments like schools and dormitories.

### Case 3

H.Ç. is a 38-year-old single man who works as a business manager in a private company. H.Ç. sees eating as a task that needs to be fulfilled only to meet his physical needs. As he is busy throughout the day, he keeps mealtimes to a minimum and usually eats the same type of simple and convenient food (e.g. sandwiches or instant soup). He does not look for taste or variety when eating; “I just want it to recharge my energy,” he says. H.Ç. also avoids eating at social events; he prefers to eat a quick meal alone at his desk rather than eating with his coworkers.

H.Ç., who has avoidant attachment, avoids emotional bonding due to his early experiences and does not have a meaningful connection with food. For her, food is just an obligation and a physical necessity. He is completely indifferent to the sensory aspects of food, such as taste, smell or visual aesthetics. Sedatic hunger may here provide an appropriate framework to describe H.Ç.‘s distant and mechanical relationship with food.

### Case 4

D.B. is a 27-year-old woman, university graduate and freelance graphic designer. D.B. experienced both physical and emotional neglect in her childhood, which negatively affected her emotional regulation skills. In her daily life, she often forgets to eat and can go for long hours without food. When she realizes that she needs to eat, instead of spending time in the kitchen, she quickly passes the meal with a pretzel or a carton of milk. For D.B., food is not an act that provides taste or satisfaction; “Eating feels like a waste of time,” she says. However, on some days, when she is under stress, she may experience a sudden increase in appetite and start eating randomly, but this does not bring any pleasure or satisfaction. She often feels guilty or empty after eating.

With disorganized attachment, D.B.‘s relationship with food is both chaotic and functional. She does not see eating as a pleasure or social experience; it is a task for her. The emotional turmoil stemming from her childhood traumas is reflected in her eating habits, completely detaching her connection with food from emotionality. Sedatic hunger may offer an appropriate conceptual framework to explain D.B.’s disconnected, indifferent, and disordered relationship with food.

### Development and Future Validation of the Sedatic Hunger Scale (SHS)

As part of our efforts to further explore Sedatic Hunger, we have initiated the preliminary development of the Sedatic Hunger Scale (SHS). This scale aims to quantify the extent to which individuals experience hunger detached from sensory or psychological engagement. While the SHS is still in its early stages, we plan to detail its development and validation in a subsequent paper. At present, item generation and expert reviews are underway, and no pilot data has yet been collected. However, illustrative examples of candidate items include statements such as “I eat without noticing the taste of my food” and “For me, eating is just a routine task. By operationalizing Sedatic Hunger through this scale, we aim to facilitate future research and clinical applications.

## Conclusion

Sedatic Hunger represents a previously unclassified form of hunger, defined by a purely functional, biologically driven motivation to eat. Unlike physiological or hedonic hunger, it is devoid of sensory engagement, emotional satisfaction, or psychological reward. This review introduces Sedatic Hunger as a unique concept, supported by clinical examples and theoretical comparisons that distinguish it from other eating behaviors.

Recognizing Sedatic Hunger has critical implications for nutrition science, especially in clinical settings involving depression, dietary monotony, or food insecurity. The development of the Sedatic Hunger Scale (SHS) marks a foundational step toward operationalizing this concept and assessing its prevalence and impact. Future research should explore its potential state-trait continuum, underlying biological and psychological mechanisms, and cultural manifestations.

In acknowledging Sedatic Hunger, we broaden the scope of how hunger and eating behaviors are conceptualized, advocating for more inclusive and personalized approaches in dietary counseling, behavioral assessment, and public health nutrition.

### Key References

- Stevenson, R. J. (2024). The psychological basis of hunger and its dysfunctions. *Nutrition Reviews*, *82*(10), 1444–1454.

° This recent article offers a deep theoretical analysis of hunger’s psychological underpinnings. It supports the foundation of Sedatic Hunger as a biologically driven, emotionally neutral construct by addressing how dysfunctions in hunger regulation affect eating behavior.

**•** Zhang, X., Ravichandran, S., Gee, G. C., Dong, T. S., Beltrán-Sánchez, H., Wang, M. C.,… & Gupta, A. (2024). Social isolation, brain food cue processing, eating behaviors, and mental health symptoms. *JAMA Network Open*, *7*(4), e244855-e244855.

° This article reveals how social isolation and mental health symptoms impact brain responses to food cues, resulting in a disconnect from sensory aspects of eating—key to understanding the emergence of Sedatic Hunger.

**•** Dakin, C., Finlayson, G., & Stubbs, R. J. (2024). Exploring the underlying psychological constructs of self-report eating behavior measurements: Toward a comprehensive framework. *Psychological Review*.

° This paper addresses the psychological constructs behind eating behavior assessments. It is highly relevant to the development and validation of the Sedatic Hunger Scale (SHS) proposed in the manuscript.

## Contributions of Sedatic Hunger To the Literature and Suggestions for Future Research

As our exploration of eating behaviors continues to evolve, the introduction of Sedatic Hunger represents a significant step forward in understanding the diverse motivations behind why we eat. This article has sought to expand the boundaries of current nutrition science by proposing a new, inclusive framework that acknowledges the existence of hunger unmotivated by pleasure or satisfaction. Sedatic Hunger sheds light on a dimension of eating that transcends the traditional models of physiological and hedonic hunger, presenting a purely functional drive to eat. By doing so, it offers a broader perspective on human eating behaviors, inviting the field to consider how individuals who engage in such indifferent or duty-driven eating perceive food as fuel rather than as a source of enjoyment [14, 35, 38, 39, 62–64].

It is important to consider that Sedatic Hunger may, in some individuals, evolve into a habitual behavior influenced by both external and internal factors. Chronic exposure to conditions such as prolonged food insecurity, persistent stress, or lifestyle constraints might reinforce this functional approach to eating, leading to a sustained detachment from sensory engagement. Furthermore, psychological traits such as anhedonia, personality predispositions, or specific coping mechanisms may interact with environmental factors to contribute to this transition.

Future research should explore this state-to-trait continuum to determine whether Sedatic Hunger can become a stable behavioral pattern and to identify the underlying mechanisms driving this shift. Longitudinal studies and clinical observations will be particularly valuable in clarifying how environmental, psychological, and social factors influence the persistence of Sedatic Hunger. Additionally, experimental designs that manipulate environmental conditions or evaluate the role of psychological traits could help disentangle the interplay between state-driven and trait-driven components of Sedatic Hunger. Investigating diverse populations and contexts will be essential to fully understand the variability and implications of this phenomenon.

### A New Perspective

Defining and analyzing Sedatic Hunger has significant implications for both theoretical and practical applications within nutrition science. By introducing this hunger type, we offer a more nuanced and inclusive approach that encompasses eating behaviors not driven by pleasure, preference, or emotional engagement. Sedatic Hunger provides a valuable conceptual tool for examining those who perceive food strictly as a biological necessity, thus challenging existing assumptions about the universal appeal of sensory satisfaction in eating [65]– [66]. This framework allows for a more comprehensive approach to dietary studies, potentially reshaping how nutrition science addresses the spectrum of human-food interactions.

### Call for Further Research

To facilitate the study of Sedatic Hunger, we have initiated the development of the Sedatic Hunger Scale (SHS), an assessment tool designed to measure this unique hunger type and quantify its presence across different populations. Future research could employ this scale to explore the biological and psychological underpinnings of Sedatic Hunger and its implications for metabolic processes, psychological well-being, and dietary habits. Investigating how Sedatic Hunger affects nutritional intake and health outcomes could yield valuable insights, especially in clinical settings. Additionally, by examining the prevalence of Sedatic Hunger and its correlation with various health parameters, researchers can deepen their understanding of this concept and build a robust foundation for more personalized approaches in dietary interventions and nutritional counseling [67–69].

The concept of Sedatic Hunger opens new avenues for exploring eating behaviors that go beyond traditional pleasure-seeking or physiological needs. Future research can investigate the biological, psychological, and social factors underlying this phenomenon and its potential implications for metabolic health, psychological well-being, and dietary practices. Understanding the prevalence of Sedatic Hunger across different populations and its role in shaping nutritional intake and health outcomes can inform personalized approaches in dietary interventions and nutritional counseling.

To support these efforts, we have initiated the preliminary development of a tool to measure Sedatic Hunger, which will be detailed in a subsequent study. This tool is expected to facilitate future research and enhance the applicability of Sedatic Hunger in both clinical and academic settings.

In conclusion, Sedatic Hunger provides a fresh perspective within the realm of nutrition science, advocating for a comprehensive approach to studying eating behaviors that extend beyond pleasure-seeking and physiological needs. By recognizing and studying Sedatic Hunger, and through tools like the Sedatic Hunger Scale, we move closer to understanding the full spectrum of human motivations for eating, paving the way for future research that enriches our knowledge and informs inclusive practices in nutrition science.

## Data Availability

No datasets were generated or analysed during the current study.
